# Advances in Peptide-Based Hydrogel for Tissue Engineering

**DOI:** 10.3390/polym15051068

**Published:** 2023-02-21

**Authors:** Negar Bakhtiary, Behafarid Ghalandari, Farnaz Ghorbani, Swastina Nath Varma, Chaozong Liu

**Affiliations:** 1 Institute of Orthopaedic & Musculoskeletal Science, University College London, Royal National Orthopaedic Hospital, Stanmore HA7 4LP, UK; 2 State Key Laboratory of Oncogenes and Related Genes, Institute for Personalized Medicine, School of Biomedical Engineering, Shanghai Jiao Tong University, Shanghai 200030, China; 3 Institute of Biomaterials, Department of Materials Science and Engineering, University of Erlangen-Nuremberg, Cauerstraße 6, 91058 Erlangen, Germany

**Keywords:** peptide-based materials, peptide-based hydrogels, self-assembly, peptide sequence, tissue engineering

## Abstract

The development of peptide-based materials has emerged as one of the most challenging aspects of biomaterials in recent years. It has been widely acknowledged that peptide-based materials can be used in a broad range of biomedical applications, particularly in tissue engineering. Among them, hydrogels have been attracting considerable interest in tissue engineering because they mimic tissue formation conditions by providing a three-dimensional environment and a high water content. It has been found that peptide-based hydrogels have received more attention due to mimicking proteins, particularly extracellular matrix proteins, as well as the wide variety of applications they are capable of serving. It is without a doubt that peptide-based hydrogels have become the leading biomaterials of today owing to their tunable mechanical stability, high water content, and high biocompatibility. Here, we discuss in detail various types of peptide-based materials, emphasizing peptide-based hydrogels, and then we examine in detail how hydrogels are formed, paying particular attention to the peptide structures that are incorporated into the final structure. Following that, we discuss the self-assembly and formation of hydrogels under various conditions, as well as the parameters to be considered as critical factors, which include pH, amino acid composi- tion within the sequence, and cross-linking techniques. Further, recent studies on the development of peptide-based hydrogels and their applications in tissue engineering are reviewed.

## 1. Peptide-Based Materials

A revolutionary era of nanotechnology has been ushered in by developing tools and techniques capable of producing, characterizing, and manipulating materials with high precision and resolution [1,2]. Bottom-up nanofabrication replaces top-down techniques constrained by the starting material’s bulk properties. Self-assembly supplies molecular nanotechnology with an attractive option for top-down miniaturization and bottom-up nanofabrication [3,4]. A self-assembling system consists of interconnected, integrated, and ordered operational systems that grow hierarchically, similar to how living systems grow. Self-assembling biomolecules, as an emerging field of bionanotechnology, enable the development of functional devices with nanoscale-ordered components and templates [5,6]. Peptide-based materials are among the most cutting-edge biomaterials that are derived from self-assembly [7,8,9]. The ability of peptides to self-assemble makes them attractive to researchers, especially as molecular building blocks [10,11]. Peptides can be synthesized using standard chemical methods, eliminating the complexity involved in synthesizing large proteins. Furthermore, they are able to control self-assembly based on physicochemical parameters, such as light, pH, strength, ionic solvent, or temperature [12,13,14].

**Figure 1:**
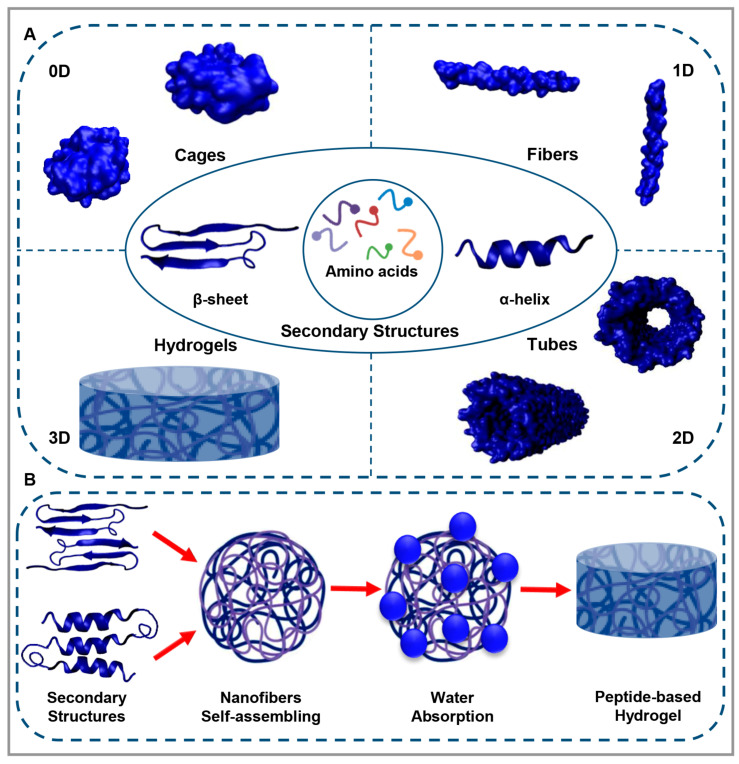
(**A**). Peptides self-assemble into different 0D, 1D, 2D, and 3D structures. (**B**). Peptide-based hydrogel formation.

In the case of self-assembling peptides, the sequence of amino acid and chemical group binding can be modified to customize the assembly characteristics [15,16]. Considering the diverse properties of amino acids, such as their electrical charge, hydrophobicity, size, and polarity, they prove to be excellent building blocks for peptide-based materials with many potential applications [17]. It is generally evident that the mechanism of assembling peptide sequence is determined by noncovalent intermolecular interactions such as electrostatic, hydrophobic, van der Waals, hydrogen bonds, and π-π stacking after the folding of the sequence into the secondary structures by the formation of α-helix and β-sheet [18,19,20]. It should be noted, however, that there are some cases in which covalent bonds such as disulfide bonds, isopeptide bonds, and metal coordination play the main role in the formation of self-assembled structures [21,22,23]. There are various structures of peptide-based materials in 0-dimensional (0D), 1D, 2D, and 3D, all of which develop by self-assembly, producing a variety of shapes including cages, fibers, tubes, and hydrogels [24,25,26], respectively, as illustrated in Figure 1A.

In nature, cages are 0D structures with a dot-like shape and are nanometers in size [27]. Polypeptide cages perform various physiological functions in living organisms, such as deoxyribonucleic acid (DNA) transport and storage, iron storage, cellular interactions, nucleic acid protection from oxidative damage, controlling protein aggregation, enzyme encapsulation, and endocytosis [28,29]. Several groups have attempted to engineer cage-like nanomaterials based on the spherical shape and function of natural nanocages and they have been applied to various applications, including drug delivery, vaccine development, and synthetic biology [28,30,31]. It should be noted that physicochemical conditions and the ratio of positively and negatively charged amino acids determine the morphology and size of cage structures [32]. The cage structure has three architecturally significant advantages. First, symmetry in the structure leads to desired interactions within the biological network. Second, the outer and inner surfaces, as well as the inter- and intra-subunits interfaces, are clearly defined, making it possible to connect fragments in a chain or separate fragments to form a functional area. Third, nanocages are very robust and can withstand many genetic and chemical modifications [29].

The use of nanofibers as 1D structures has been considered for biomedical applications as well [33,34]. Nanofibers provide unique templates for controlling the molecular packing and internal order of therapeutic delivery devices so that they can interact effectively with cells and tissues in a controlled and predictable manner [35,36]. The use of nanofibers, which have the major content of β-sheets, has emerged as an effective tool for biomedical applications in recent years [35,37]. Furthermore, helix bundles have been used to design self-assembling nanofibers. They have typically sticky-ends that affect helix further interaction, so leaving dangling sticky-ends initiates the addition of helices at both ends and supports growth [38]. Peptide nanofibers have allowed researchers to model the self-assembling process of amyloid fibrils and study the biological process of their formation, which is an essential step toward studying the relevant diseases [39,40].

Nanotubes as 2D structures can be used in numerous applications, including drug delivery, catalysis, chemotherapy, electronics, and molecular separation. Several methods have been applied to prepare noncovalently self-assembled nanotubes, notably helical structures, rod-like hollow bundles, and stacked rings [41,42,43,44]. Due to their hollow structure, nanotubes can bind small molecules, demonstrating their potential for drug delivery [45]. Diphenylalanine (FF) peptide is the simplest building block for self-assembly into peptide-based materials, forming a long tube-like structure (about 100 µm). This structure is formed by hydrogen bonds and π-π stacking during the self-assembly process [46]. In recent years, cyclic peptide nanotubes, in which π-π stacking and hydrogen bonds are the main driving forces for their formation, have attracted much attention [47]. A nanotube using this self-assembly adopts a specific orientation in which the side chains of the amino acids face the exterior while the amino and carbonyl groups on the backbone face perpendicular to the ring and form hydrogen bonds [48]. Additionally, some nanotubular structures are generated through the self-assembly of peptide amphiphiles (PAs) [49].

Although these self-assembling peptide architectures are highly promising, their structures are not sufficient for specific interactions with cells. Hence, the next logical step would be to pair biologically active and functional peptides directly to create scaffolds capable of interacting with cells and tissues [50]. A peptide-based hydrogel is a highly hydrated, 3D structure composed of peptide-based nanofibers that absorb water around them by joining together and forming interwoven networks (Figure 1B) [51]. Peptide-based hydrogels are attractive biomaterials that can be used in 3D stem cell cultures, regenerative medicine, tissue engineering, bioprinting, sustained release, the acceleration of diabetic wounds and skin wounds, as well as immediate hemostasis [52,53].

Tissue engineering requires a 3D environment for cell growth, differentiation, and, ultimately, tissue formation, and these hydrogels have achieved excellent results in simulating this environment [54,55]. Currently, hydrogels are used in the biomedical and pharmaceutical fields for various purposes, including drug delivery systems, wound dressings, tissue engineering, and contact lenses. Hydrogels derived from natural sources have been the subject of recent research. A variety of natural-based materials can be fabricated into hydrogels, including proteins [56], polymers [57,58], and peptides. The advantages of peptide-based hydrogels make them an attractive material for tissue engineering and biomedical applications. The advantages and disadvantages of each group are compared in Table 1. Accordingly, the use of peptide-based hydrogel is expected to promote cell survival and growth, as well as direct cell behavior. The peptide-based hydrogels’ high water content facilitates the diffusion of growth factors, nutrients, and biochemical signals, allowing cells to migrate and grow similarly to biological tissues. [59]. In addition, the sol-gel conversion does not involve harmful chemicals, and the gel-degraded products can be metabolized by the cells. As a result of the physiologically induced sol-gel transition, the hydrogel’s high internal hydration, and the presence of nanofibers, peptide-based hydrogels can potentially interact with cells for tissue engineering. The physicochemical properties of peptide-based hydrogels make them ideal for nanomedical applications since they are easy to handle, non-immunogenic, non-toxic, biodegradable, non-thrombogenic, and can be used in localized therapy [60,61]. Furthermore, this class of biomaterials can help overcome challenges associated with the assessment of cells and tissues [62].

## 2. Peptide-Based Hydrogels

Generally, peptide-based hydrogels consist of nanofibrous networks interconnected by chemical or physical bonds. Among the key characteristics of these materials are their mechanical stability, microporous structure, biocompatibility, high water content, tissue-like elasticity, and injectability. Further, peptide-based hydrogels can easily be developed by modifying the binding of the amino acid side chains and incorporating desired amino acids into the backbone to meet specific requirements [70]. A non-covalent self-assembly process can be used to produce hydrogelation under the appropriate conditions, so that nanofibers are formed in fiber-based hydrogels by self-assembly [71]. The thickness and length of these fibers increase as they elongate in 3D, ultimately forming fibrillar networks. In this manner, water can be entrapped within these complex networks of peptides, resulting in a self-supporting hydrogel [72]. Synthesizing a biomimetic physical hydrogel capable of flowing and recovering after shearing is an example of non-covalent interaction to form peptide-based hydrogels. Shear recovery can facilitate minimally invasive procedures and bioprinting-based tissue engineering. In tissue engineering, stem cell transplantation is often associated with poor cell survival. Due to their properties, these hydrogels are able to be injected directly from the needle and increase cell viability by reducing the mechanical forces that destroy the cell membrane [73]. Peptide-based hydrogels require the incorporation of adhesion peptides to mimic the extracellular matrix (ECM); RGD is the most commonly used peptide sequence for this purpose [74].

Peptide-based hydrogels consisting primarily of antiparallel β-sheets form an architecture in which non-polar and polar amino acids appear in an alternating fashion. The non-polar amino acid seems to be responsible for the strength of β-sheet-rich fibers in these systems, whereas the polar amino acid appears to be responsible for the interactions between fibers and aggregation. When fibers reach a critical concentration, they entangle and form a 3D network that eventually forms a self-supporting hydrogel [75]. On the other hand, intermolecular interactions mediated by electrostatic and hydrophobic interaction as well as hydrogen bonding stabilize helix bundles that can then be assembled into fibers and hydrogels. Depending on pH and salt levels, charged α-helix peptides can assemble into various micro- and nanoscale structures [76]. Helix bundle assembly is significantly more complex than β-sheet assembly, in which peptides with only two amino acids can be assembled into fibers. However, these longer α-helix sequences offer many opportunities to attach fluorescent labels and reactive groups in addition to precisely controlling binding strength and, in particular, stimulus-response, assembly order, and stoichiometry [77].

Charge, ion, and pH profoundly affect the peptide structures within peptide-based hydrogels [78]. Xing et al. [79] observed that β-sheets change into α-helix under alkaline conditions within the self-assembled fluorenylmethoxycarbonyl (Fmoc)-FF nanofibers. The C-terminus of Fmoc-FF becomes charged when deprotonated under alkaline conditions. The charges on carboxyl groups (COOH) have been found to cause an increase in β-sheet content when deprotonated in Na_2_B_4_O_7_-EDTA buffer at pH 8.5.

As well, the sequence of peptides plays a significant role in determining the structure of peptide-based hydrogels. In this regard, a total of four octapeptides consisting of F, E, A, and K in an alternating fashion with charge have been developed by Saiani and coworkers [80]. Sequences with phenylalanine were found to produce self-supporting hydrogels more readily, whereas sequences containing alanine could only produce viscous solutions even when high concentrations were used. There was an increased tendency for peptides with higher amounts of alanine and glutamic acid residues to form α-helical structures (AEAKAEAK and AEAEAKAK sequences), compared to those with lower amounts of glutamic acid and alanine residues, while two other peptides with FEFKFEFK and FEFEFKFK sequences developed β-sheet structures.

The stiffness of hydrogels needs to be finely tuned in tissue engineering because cells respond to stiffness in their microenvironment and change their properties. Ideally, the matrix encapsulating cells should match the stiffness of the cells’ natural environment to achieve a more realistic tissue culture study. Various factors affect the mechanical properties of peptide-based hydrogels, including peptide sequence, peptide concentration, peptide hydrophilicity or hydrophobicity, amino acid charge, sequence length, pH, temperature, electrolyte concentration, electrolyte ion types, and ion size. The parameter ranges can be easily controlled, which allows peptides to be utilized in the design of tailor-made hydrogels. Nevertheless, there is a need to develop the stiffness of the peptide-based hydrogels since they are not as stiff as hard tissues. Hence, various techniques are developed to improve the stiffness of biocompatible peptide-based hydrogels [60].

The ability to control the architecture of peptide-based hydrogels with external stimuli is essential in tissue engineering applications. In many applications [81,82,83,84,85], a precursor bio-fluid is injected into areas that need regeneration and converted to a gel in situ. External physical and chemical stimuli, such as temperature, pH, or special additives, are highly effective at creating the scaffold and completing the process. An enzymatic trigger is one of the most popular external stimuli. Several advantages of peptide-based hydrogelation triggered by enzymes include biocompatibility, homogeneity, and mild reaction conditions. Several experiments have been conducted by Ulijn et al. [86] using proteases for reverse hydrolysis to catalyze the self-assembly of peptide-based hydrogels. As a result of coupling two amino acids using Thermolysin, which facilitates the formation of peptide bonds thermodynamically, hydrogels were formed. Reverse hydrolysis has the advantage of producing no by-products other than water. The team developed a series of alkaline phosphatase (ALP)-responsive PAs based on Fmoc-protected dipeptides [87]. As well, enzymatic catalysis enables peptides to self-assemble into hydrogels that are highly effective antimicrobial agents. Moreover, the researchers found that ions had a significant effect on the network structure of the enzyme, and these changes could be used to control self-assembled peptide nanostructures. This resulted in different nucleation and growth rates for self-assembled nanostructures, leading to differences in mechanical properties and chirality [88]. Yang et al. [89] reported that Tyrosinase could promote a transition of gel-sol in Ac-YYYpT-OMe hydrogel. The enzyme-responsive peptide sequence can assemble into hydrogels and disassemble by Tyrosinase when exposed to it. As a result of the overexpression of Tyrosinase in malignant melanoma cells, this enzyme-responsive hydrogel can encapsulate anticancer drugs. According to Yang’s group, enzymes promote peptide self-assembly to facilitate the folding into the α-helix conformation [90]. ALP catalysis can convert CRB-GDFDFpDY peptides to CRB-GDFDFDY, resulting in helix-shaped hydrogels. Compared to hydrogels with a sheet conformation, this exhibited superior integrity against proteinase K degradation. Several studies have confirmed the increase in helix content in the presence of enzymes [91,92].

Aside from the numerous advantages peptide-based hydrogels possess, their poor mechanical properties and low elasticity present limitations in some applications, which can be overcome by cross-linking appropriately. Peptide-based hydrogels are stiffer compared to other peptide-based materials due to the chemical cross-linking within the structure or between the peptides and other macromolecules. Liyanage et al. [69] demonstrated that covalent cross-linking of peptide–chromophore–peptide triple sequences enhances the mechanical stability of self-assembled peptide-based hydrogels through their incorporation with polyethylene glycol (PEG)-based small guest molecules [69]. The effects of azide–alkyne cycloaddition amine-reactive cross-linking, enzymatic cross-linking, and thiol-ene reaction methods were investigated. It was found that the hydrogels’ viscoelastic properties varied significantly as a result of the various cross-linking reactions. Despite this, no clear relationship was found between the different cross-linking methods and the final rigidity of the hydrogel. According to Ding et al. [93], the mechanical strength of FmocFFGGGY peptide-based hydrogel increased due to photo-cross-linking. A well-established ruthenium mixture (Ru(bpy)3Cl2 catalyzes tyrosine transformation to dityrosine during light irradiation. First, the rationally designed peptides are assembled into hydrogels based on the geometric constraint of amino acids and their hydrophilicity. Then, the cross-linking mechanism is activated with white light within 2 min of adding Ru(bpy)3Cl2. Cross-linked hydrogels with a storage modulus of about 100 kPa exhibit 10^4^-fold better mechanical stability than non-cross-linked hydrogels due to densely interwoven fibrillar networks of peptide dimers linked by dityrosine links. They emphasized the following factors to implement this approach: (1) Geometrical and hydrophilic constraints need to be considered when designing the peptide sequence to avoid self-assembly and cross-linking interference. (2) In situ photo-cross-linking is necessary for hydrogels to ensure the adequate co-assembly of cross-linked and uncross-linked peptides.

Overall, covalently cross-linked hydrogels can deform elastically while maintaining their mechanical strength. Nevertheless, the lack of suitable porous structures for the diffusion of large substrates, an essential requirement for cell culture and biocatalysis, continues to pose a challenge. A combination of peptide-based hydrogels and polymeric materials can achieve both advantages as a physical cross-linking [94]. In contrast to chemical cross-linking, this technique enables the direct fabrication of hydrogels from their native building blocks without requiring any chemical modifications or harsh chemical reactions. However, hydrogels that contain physically cross-linked chains do not undergo a notable change in volume during the sol-gel transition. These materials can be produced using self-assembling polymers, which are triggered by external factors such as pH and temperature. When an external stimulus is applied to a polymer-peptide solution of low viscosity, it can rapidly form a polymer-peptide hydrogel. Physical cross-linking generally constructs weaker peptide-based hydrogels than chemical cross-links, causing them to be more sensitive to mechanical stresses [95].

The advantage of physically cross-linked hydrogels is to use them as injectable materials. As a result of physical cross-linking, most organic solvents and cross-linking reagents are not necessary, making these hydrogels suitable for biomedical applications such as drug delivery and tissue engineering [95]. Accordingly, Xing et al. [96] developed a robust hydrogel by physically cross-linking poly-L-lysine modified with the mercapto group (PLL-SH) and Fmoc-FF. By entangling the negatively charged dipeptide Fmoc-FF with the positively charged PLL-SH, electrostatic interactions result in injectable hydrogels formed through the entanglement of the peptide nanofibers. PLL-SH modified with a mercapto group (-SH) creates disulfide bonds that contribute to the mechanical strength of the hydrogel. The peptide-based hydrogel could be squeezed with a 26-gauge needle without causing any blockages. According to the rheological analysis of the Fmoc-FF/PLL hydrogel, it has more acceptable properties than the individual Fmoc-FF hydrogels.

Consequently, by absorbing water, nanofibrous structures are changed into peptide-based hydrogels. During the elongation of fibers in 3D, their thickness and length increase, eventually forming fibrillar networks. As a result of these complex networks of peptides, a self-supporting hydrogel is formed. Hydrogels containing the predominant contribution of β-sheets have higher mechanical properties, and those containing the majority of α-helixes have better biological properties. The structural content of peptide-based hydrogels can be modified by controlling variables such as peptide sequence, concentration, charge, and pH, and by adjusting these structures, the properties of the final hydrogel can be altered. In order to use peptide-based hydrogels in biomedical applications, they need to be chemically or physically cross-linked [97]. The physical cross-linking in the form of polymer–peptide interaction provides more favorable properties for tissue engineering.

## 3. Peptide-Based Hydrogel in Tissue Engineering

In addition to drug delivery [98] and wound healing [96,97], tissue engineering is a rapidly expanding area of biomaterial science committed to restoring and replacing damaged tissues. In spite of significant advances in tissue engineering technology, the development of effective biocompatible 3D scaffolds still remains a challenge [98]. The ease of use and customization of self-assembling peptide-based hydrogels make them useful for tissue engineering [99]. Natural or synthetic amino acids can be used to produce them, as well as polymerization techniques. In biomimetic 3D environments, peptide-based hydrogels are valuable for studying cells in vitro and facilitating tissue regeneration in vivo. In order to control adhesion, morphology, and the expression of proteins, peptide-based hydrogels can be utilized to construct microenvironments [62]. A wide range of tissues can be regenerated using peptide-based hydrogels, including nerve, muscle, cartilage, bone, and skin. This section discusses recent research on the engineering of each of these tissues, which are summarized in Table 2.

### 3.1. Nerve

**Figure 2:**
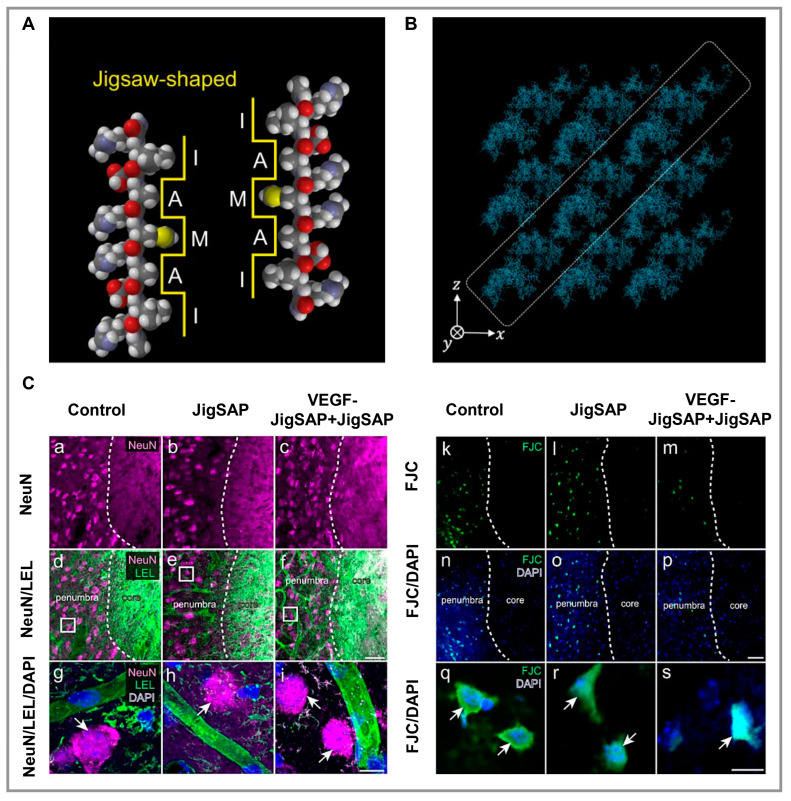
(**A**). Space-filling model of the JigSAP sequence, demonstrating the jigsaw-shaped hydrophobic surfaces. (**B**). Illustration of JigSAP supramolecular structures in water obtained by all-atom molecular dynamics simulation. (**C**). (**a**–**f**) neuron marker NeuN in magenta color (**a**–**c**) and NeuN/endothelial cell marker Lycopersicon Esculentum lectin (LEL) in magenta/green stained (**d**–**f**) images of the border between the injured core and the penumbra. (**g**–**i**) Magnified views of NeuN (white arrows), LEL, and DAPI stained with magenta, green, and blue, respectively. (**k**–**p**) Fluoro-Jade C (FJC) with green (**k**–**m**) and FJC/DAPI (green/blue) (**n**–**p**) images of the border between the injured core and the penumbra. (**q**–**s**) Magnified views of FJC/DAPI (green/blue) in the penumbra (white arrows). Reproduced under the terms of the Creative Commons Attribution 4.0 International (CC BY 4.0) license. [103] Copyright 2021, Nature.

Chai and colleagues examined an IKVAV peptide hydrogel scaffold consisting of PNIPAM-b-poly(AC-PEG-COOH) and PNIPAM-b-poly(AC-PEG-IKVAV). They determined whether it promoted angiogenesis, inhibited keratinocyte differentiation, prevented their adhesion, and reduced the formation of glial scars [100]. Through this scaffold, angiogenic factors were expressed, pro-inflammatory factors were decreased to a certain level, glial scar tissue regrowth was inhibited, and damaged tissue was repaired, making it appropriate for tissue engineering applications in the central nervous system (CNS). An artificial neurovascular microenvironment was constructed by Zhang and colleagues using a dual-functionalized nanofiber peptide-based hydrogel including RGI and KLT, which are mimetic peptides of vascular endothelial growth factors (VEGF) and brain-derived neurotrophic factors [101]. In order to synthesize RGI and KLT, RGIDKRHWNSQ and KLTWQELYQLKYKGI bioactive motifs were added to the C-terminus of RADA (Ac-RADARADARADARADA-NH2). Based on in vitro results, this nanofibrillar peptide-based hydrogel enabled pheochromocytoma (PC12) cells to outgrow and improved the tube-like formation in human umbilical vein endothelial cells (HUVECs). According to in vivo studies using rat brain injury models, this hydrogel facilitates neurovascular crosstalk by modulating paracrine factors secreted by PC12 cells and HUVECs. Co-culturing these cells on this hydrogel significantly enhanced their interaction, resulting in an accelerated differentiation of PC12, which resulted in an increase in pseudopodia and tube-like structures in HUVECs. Hivare and colleagues chemically conjugated the IKVAV peptide to a DNA-based hydrogel coated on glass [102]. Sulfo-MBS (*m*-maleimidobenzoyl-*N*-hydroxysulfosuccinimide ester) crosslinking was used to decorate native DNA hydrogels using peptides linked to single oligo primer strands. The IKVAV peptide was synthesized chemically to stimulate neural differentiation and attachment. Neuroblastoma stem cells treated with this hydrogel had a more significant differentiation of the cytoskeleton and microtubules of neurons. Additionally, they had longer neurites and modified endocytic mechanisms compared with those treated with the unmodified DNA hydrogel. Yaguchi and coworkers designed a peptide-based hydrogel called JigSAP that includes the AXXXA glycophorin A (GYPA) motif and is inspired by the dynamic self-assembling characteristics of natural sequences in order to create an artificial ECM for brain regeneration [103]. The sequence of JigSAP is Ac-RIDARMRADIR-NH2 (Figure 2A,B), which consists of hydrophilic and hydrophobic amino acids, resulting in an amphiphilic structure with exposed hydrophilic and hydrophobic surfaces in the β-sheet. A peptide-based hydrogel exhibited a controlled release of VEGF, and in vivo studies on a mouse stroke model revealed cell transplantation-free regenerative capabilities, indicating this hydrogel has significant potential for brain regeneration (Figure 2C). Wiseman et al. [104] evaluated Fmoc-DIKVAV as a scaffold for grafting cells after spinal cord injury (SCI) caused by mild thoracic contusions. As a result of the incorporation of the laminin-1 sequence IKVAV in the Fmoc-DIKVAV hydrogel, it promotes cell adhesion and growth, angiogenesis, and neurite outgrowth, as well as reducing cell death, astrogliosis, and enhancing function in SCI mice. The Fmoc-DIKVAV hydrogel was fabricated to have a gelation time of 2 s at pH 7.4 and exposed to 15 min of ultraviolet light. The hydrogel was mixed with either viable or nonviable rat mesenchymal precursor cells (rMPCs). A comparison was made between the results achieved with hydrogel, rMPC, and control with only SCI. The Fmoc-DIKVAV hydrogel scaffold helped facilitate axonal regrowth after contusion SCI. This hydrogel could be used in neuroscience and neuroregeneration as conduits for nerve tissue. Using the same IKVAV sequence and peptides from PA, the Ji group has produced bioactive supramolecular nanofibers in another study [105]. In this study, the molecules of IKVAV-PA (C16V2A2E4GIKVAV) and its scramble sequence VKIVA-PA (C16V2A2E4GVKIVA) were synthesized using a standard Fmoc solid-phase peptide synthesis procedure and purified using reverse-phase HPLC. After one week, these peptide-based hydrogels initiated the commitment to neuroectodermal lineage, and many major biological components were expressed, such as microtubule-associated protein-2 (MAP-2), b-III tubulin (TUJ-1), and neuronal nuclei (NEUN), as well as ECM laminin. A distinctive elongation of bone marrow mesenchymal stem cells (BMSCs) was observed only in cells that were in contact with supramolecular nanofiber gels. As a result of the study, it was concluded that BMSCs combined with IKVAV-PA supramolecular nanofibers could enhance the function of endogenous or transplanted BMSCs within the CNS following traumatic injury.

### 3.2. Muscle

**Figure 3:**
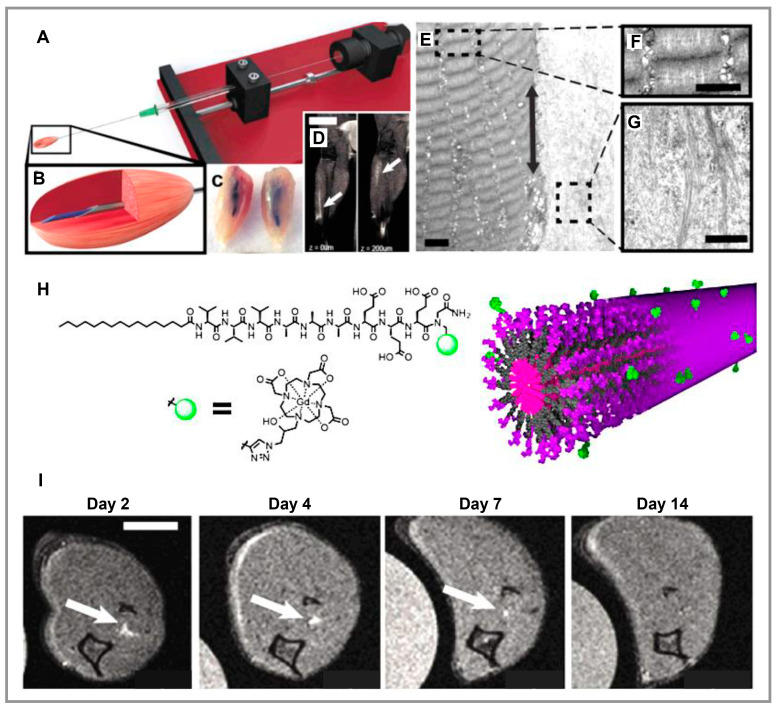
(**A**). The injection device is illustrated by the schematic depicting a Hamilton syringe on a retractable leadscrew linear actuator platform. (**B**). A zoomed-in image illustrates the injection of PA solution (the blue line) into muscle tissue. (**C**). Macroscopic view of collected samples after injection with an Evans blue-dyed mid G′ PA solution. (**D**). The results of two MRI scans taken from the same mouse leg at a distance of 200 um. In this image, white arrows indicate the presence of the PA hydrogel along the length of the muscle (Scale bar = 1 cm.) (**E**). TEM images illustrating muscle sarcomere markings next to the PA hydrogel injected. The black arrow points toward the muscle’s long axis and the PA nanofibers. (**F**,**G**). High-magnification insets illustrate the muscle and the nanofibers, respectively; the myofibers and nanofibers are oriented parallel to the muscle’s long axis (Scale bar = 1 μm.) (**H**). Illustration of the gadolinium chelate illustrated by the green sphere and PA molecule utilized for following the hydrogel in vivo and molecular graphics of the supramolecular PA nanofiber consisting of 5% Gd(III)-labeled PA and 95% C16V3A3E3 PA. (**I**). Axial MRI scan displaying the Gd-PA scaffold (illustrated by arrows) of the same leg at 8 d postinjection; the Gd-aPA is no more detectable by day 15 (Scale bar = 500 μm). Reproduced under the terms of “Freely available online through the PNAS open access option”. [109] Copyright 2017, PNAS.

The researchers designed a dual crosslinking conducting peptide-based hydrogel comprised of maleimide-modified hyaluronic acid (MaHA) and gelatin, which provided self-healing and good mechanical properties, as well as degradable according to the myocardium’s systolic-diastolic rhythm, which enhanced the thickness of the myocardium significantly [106]. A bifunctional myocardial infarction (MI)-responsive peptide sequence, GCNS-GGRMSMPV-KLTWQELYQLKYKGI (GGR-KLT) consisting of both a therapeutic KLT and a cleavable GGR sequence was developed, and further bound to MaHA through Michael addition using both MaHA maleimide and GGR sulfhydryl groups in order to stimulate angiogenesis through matrix metalloproteinase-2 (MMP 2). GGR, a peptide sequence cleavable by MMP-2 that mediates the interaction between MaHA and KLT, can regulate the release of KLT on-demand to consume overexpressed MMP-2 in damaged tissue. In an MI model utilizing this hydrogel as an encapsulation medium, BMSCs were injected, and in vivo experiments demonstrated that this system has three distinct advantages. First, there is adequate prevention of uncontrolled matrix degradation. Second, the induced release of KLT resulted in dramatic improvements in angiogenesis. Third, it improved myocardial regeneration over 28 days due to the induced release of KLT. Experiments revealed a high potential for clinical application of this hydrogel based on the complete regeneration and recovery of damaged myocardium. Using a multi-component co-assembly peptide and a reactive oxygen species (ROS) scavenging peptide, Zhan and colleagues developed a composite hydrogel composed of conducting polypyrrole (PPy) [107]. As a result of the incorporation of TEMPOL(Nap-FFK(TEMPOL)GRGD) into the hydrogel, it can scavenge ROS effectively. By engineering physical milling of the conductive PPy membrane, they created micro/nano-sized particles that adhered to self-assembly structures through binding with the T59 (Nap-FFEG-THRTSTLDYFVI) peptide, resulting in a novel ROS-scavenging/conductive composite hydrogel. This conductive and antioxidant peptide-based hydrogel effectively removed ROS from cardiomyocytes during oxygen-glucose deprivation. Based on this work, hydrogel may enhance cardiomyocytes’ contractile and electrical properties based on the demonstrations of expression of the CX-43 protein and rhythmic intracellular Ca^2+^ puffs. The combination of this hydrogel with cardiomyocytes was found to improve cardiac repair and function significantly. Alheib and colleagues developed skeletal muscle scaffolds using gellan gum (GeG) hydrogels tailored to laminin-based peptides [108]. Hence, murine skeletal muscle cells (C2C12) attached to three laminin-derived peptides, KNRLTIELEVRTC, CIKVAVS, and RKRLQVQLSIRTC. Additionally, GeG, which has been chemically functionalized, was tested for its ability to bind laminin-derived peptides. Regarding binding rates, C2C12 tethered to peptides V, T, and Q in percentages of 10%, 48%, and 25%, respectively, while peptides tethered to GeG in percentages of 60%, 40%, and 31%. Different levels of polymer content in peptide-biofunctionalized hydrogels resulted in different surface mechanics and peptide exposure. Even though all hydrogels demonstrated cellular adhesion, only those functionalized with peptide Q illustrated differentiation and spreading. A PA hydrogel scaffold was developed by Sleep and colleagues to transplant muscle stem cells [109]. PAs were synthesized on a custom peptide synthesizer using an aliphatic palmitoyl tail (C16) and a hydrophilic cap of six or nine amino acids. Afterward, they exposed the amino acid cap by entropic mixing and aggregated the hydrophobic tail by annealing PA into long-axis nanofibers randomly ordered. In order to create stable, noodle-like scaffolds that have liquid crystalline properties due to their parallel orientations to the direction of extrusion, PAs were extruded into physiological calcium concentrations in a culture medium and created a high-ordered nanofiber structure. These scaffolds were found to provide myogenic progenitor cells with a suitable biological environment for the survival, maturation, and regeneration of muscle tissue (Figure 3).

### 3.3. Cartilage

**Figure 4:**
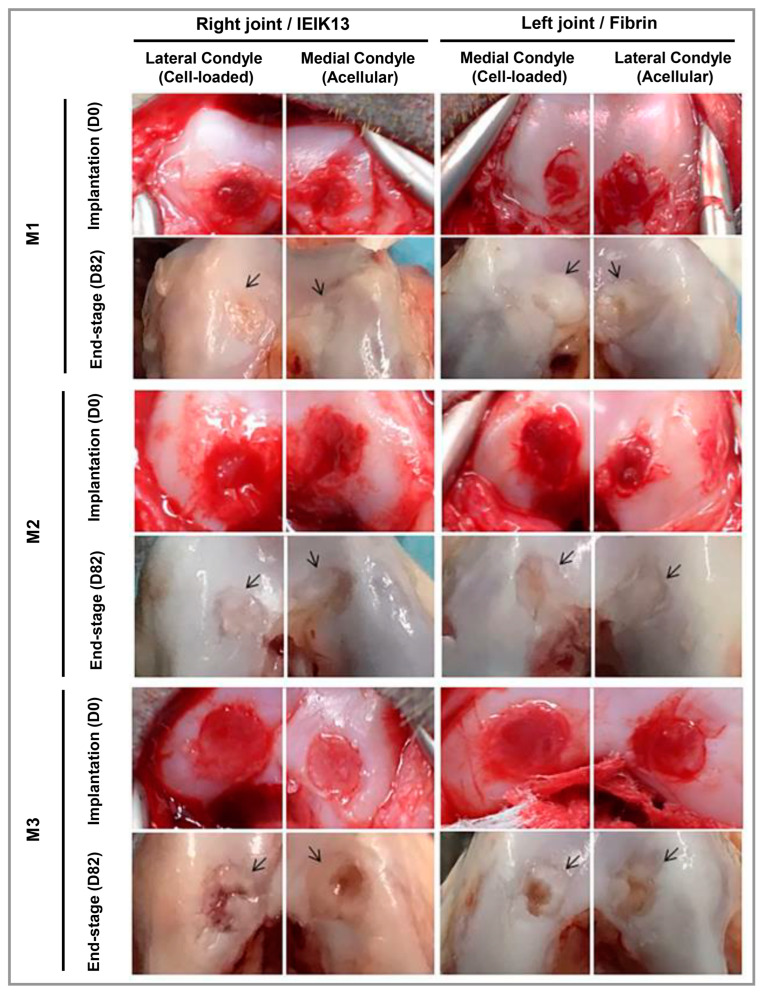
An observation of macroscopic cartilage lesions treated with IEIK13- and fibrin-based hydrogels on the implantation day (D0) and 82 days later (D82). Chondrocytes were either loaded or not loaded into the hydrogels, as indicated. A specific anatomical site for implantation in the knee joint has been identified, and M1, M2, and M3 correspond to three Cynomolgus monkeys. There is an indication of the original margin of the defect by the arrows. The central regions of the M1 and M2 defects appear depressed or filled with rough tissue, whereas the central regions of the M3 defects seem to be poorly resurfaced. Reproduced under the terms of the Creative Commons Attribution 4.0 International (CC BY 4.0) license. [112] Copyright 2021, Nature.

Ye and co-workers linked Ac-(RADA)4-CONH2 (RAD) peptide with transforming growth factor-β (TGF-β)-simulating peptide LIANAK (CM) to produce the synthetic self-assembling peptide Ac-(RADA)4-GG-LIANAK-CONH2 (RAD-CM) [110]. The final hydrogel induced chondrogenic gene expression and ECM deposition, which was subsequently combined with decellularized cartilage ECM to form a mechanically strengthened composite scaffold for the regeneration of articular cartilage. In addition to its favorable bioactivity and structural characteristics, this composite scaffold can enhance both neocartilage regeneration and osteochondral regeneration, providing a potential for in situ cartilage regeneration, as it contains CM peptides that simulate TGF-β. Thomas and colleagues developed a hydrogel with an interpenetrating dynamic network to support chondrocyte growth and differentiation [111]. They achieved this goal by self-assembling PA (NVFFAC short sequence) into nanofibers and embedding them in a chemically crosslinked polysaccharide network composed of carboxymethyl cellulose dialdehyde and carboxymethyl chitosan. Because of the hydrogel’s flexible structure, numerous cellular functions were facilitated without compromising its integrity. Human chondrocytes were stimulated to undergo in vitro chondrogenesis using hydrogels, and the authors observed enhanced cell growth and cartilage-specific ECM secretion. Dufour’s research team studied the repair of full-thickness cartilage defects in the cynomolgus monkey using an Ac-IEIKIEIKIEIKI-NH2 (IEIK13) [123] peptide sequence in conjunction with articular chondrocytes treated with a chondrogenic cocktail of bone morphogenetic protein (BMP)-2, insulin, and triiodothyronine (T3), referred to as BIT [112]. Monkey articular chondrocytes treated with BIT produced cartilage using IEIK13 hydrogel. Full-thickness cartilage injuries could be restored by implants that contained or lacked chondrocytes (Figure 4). To evaluate growth factors, Zanotto et al. [113] used a self-assembled peptide hydrogel [KLDL]_3_, with the sequence AcN-(KLDL)_3_-CNH_2_ [124], functionalized with platelet-derived growth factor BB (PDGF-BB) and heparin-binding insulin-like growth factor (HB-IGF-1) with trypsin pretreatment to examine the healing of a critical cartilage injury after rigorous exercise in horses. Better integration of newly produced cartilage was reported compared with microfracture alone. To encapsulate BMSCs and nasal chondrocytes (NCs) using PEG-peptide hydrogels functionalized with TGF-3 and BMP-2, Stüdle and colleagues applied enzymatic polymerization [114]. The hydrogels were synthesized by mixing an equal ratio of 8-armed PEG conjugated with peptides responsible for cross-linkage via transglutaminase factor XIII and cell-mediated degradation (PEG-Gln: NQEQVSPL-ERCG and PEG-MMPsensitive-Lys: FKGG-GPQGIWGQ-ERCG) at pH 7.6. The results demonstrated that BMSCs could form osteoblast-like structures by endochondral ossification, while NCs formed cartilage to maintain structural stability.

### 3.4. Bone

**Figure 5:**
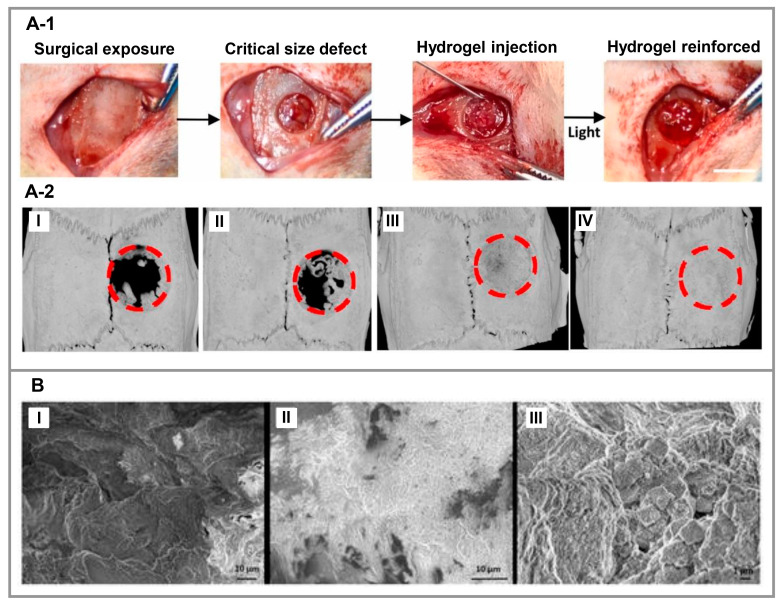
(**A-1**). Peptide-based hydrogels loaded with KP and QK synergistically enhance critical bone repair in vivo. Images of the surgical process (scale bar = 5 mm). (**A-2**). Illustrating 3D reconstruction images and the bone regeneration of the injured area following treatment with (**I**) DN-GMO hydrogel alone (GMO Ctrl) or DN-GMO hydrogel with (**II**) QK (QK/GMO), (**III**) KP (KP/GMO) and (**IV**) KP + QK(KP/QK/GMO) combined after 8 weeks. Reproduced under the terms of the Creative Commons Attribution-NonCommercial-NoDerivatives 4.0 International (CC BY-NC-ND 4.0) license. [115] Copyright 2022, Elsevier. (**B**). Images obtained by SEM of engineered tissues on DEX-loaded RADA 16-I hydrogel after 21 days of cell culture. Three morphologies that have characteristics of (**I**) cells (scale bar = 10 μm), (**II**) ECM/connective tissue (scale bar = 10 μm), and (**III**) minerals (scale bar = 1 μm) are evident. Reproduced under the terms of the Creative Commons Attribution 4.0 International (CC BY 4.0) license. [116] Copyright 2019, MDPI.

Li et al. [115] have recently designed a self-healing injectable hydrogel incorporating angiogenic peptides (QK: KIPK(Ac)ASSVPTELSAISTLY) and osteogenic peptides (KP: IPK(Ac)ASSVPTELSAISTL) that enhances vascularized regeneration of minor, irregular bone defects. The structure of this peptide-based hydrogel was produced by forming a Schiff base reaction between amino groups of gelatin methacryloyl (GelMA) and aldehyde groups of oxidized dextran (ODex), which results in enhanced injectability and shape fidelity of the final hydrogel. Results obtained in vitro from the peptide-based hydrogel demonstrated a considerable increase in the osteogenic differentiation of BMSCs and the angiogenesis capability of HUVECs. Moreover, they examined bone formation in rat calvaria and found that loaded KP and QK peptides had synergistic effects. This demonstrates that this composite hydrogel can be used as an efficient and safe scaffold for vascularized bone regeneration (Figure 5A). A peptide-based hydrogel loaded with dexamethasone (DEX) was prepared by Panek’s group and demonstrated to release DEX concentration optimally under perfusion force using 4 × 10^−4^ M DEX-loaded [COCH3]-RADARADARADARADA-[CONH2] [116]. In this study, bone tissue was regenerated on a peptide-based hydrogel scaffold for 21 days with the DEX perfusion rate of 0.1 mL/min, illustrating the potential of the system for orthopedic tissue engineering (Figure 5B). Zhang and colleagues developed a RATEA16 (RA, [CH3CONH] RADARADARADARADA-[CONH2]) hydrogel scaffold for sustained release of rhVEGF165 and BMP-2 to achieve in vitro osteogenic/vasculogenic differentiation of HUVECs and human stem cells of the apical papilla (SCAPs) [117]. It was concluded that RATEA16 could be an effective clinical strategy in tissue engineering, especially in bone reconstruction, due to its ability to deliver growth factors quickly and precisely. Based on the two previous studies, in the field of bone tissue, a slight change in the peptide sequence RADARADARADARADA-[CONH2] can release additives or create a suitable substrate for cell growth and function. Using two tetrapeptide hydrogel scaffolds described by Alshehri and colleagues, hMSCs were grown and differentiated into osteogenic cells [118]. The peptide sequences Ac-Ile-Val-Phe-Lys-NH2 (IVFK) and Ac-Ile-Val-Cha-Lys-NH2 (IVZK) were synthesized using solid-phase peptide synthesis (SPPS)44, and their purification was performed via mass spectrometry. In solidified form, tetrapeptides can form hydrogels with properties similar to the ECM and provide cells with a 3D environment with appropriate mechanical properties. In these hydrogels, positively charged lysine amino acids affect cell adhesion and spreading. Halperin-Sternfeld and colleagues reported that HA combined with an ultrashort aromatic peptide-based hydrogel can stimulate bone regeneration [119]. A nanofibrous FmocFF/HA hydrogel with an average storage modulus of 46 kPa promoted osteogenic differentiation and calcium synthesis in MC3T3-E1 preosteoblasts. Implantation of the hydrogel in vivo resulted in the restoration of approximately 93% of bone mass. In addition, bony islets developed along the entire defect, not just along its margins. The periosteum-hydrogel interface was lined with elongated macrophages within a week of implantation, and three weeks later, the macrophages were dispersed throughout the newly formed bone tissue.

### 3.5. Skin

**Figure 6:**
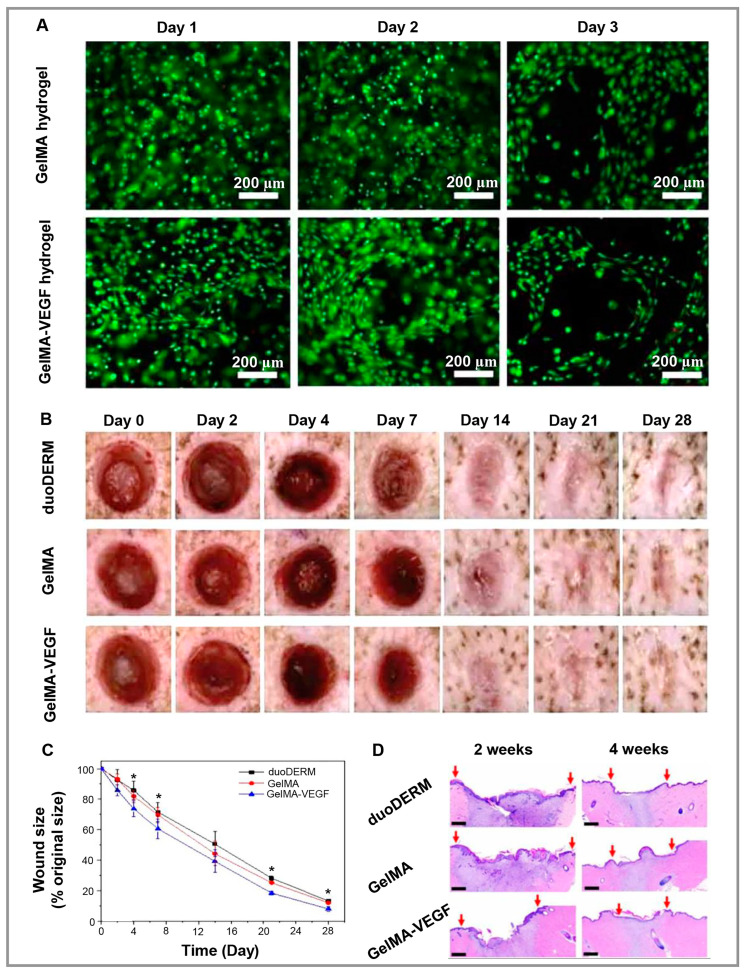
(**A**). The tubular structure formed by GelMA and GelMA-VEGF hydrogels in the presence of HUVEC cells. Live/Dead assay images of HUVECs. There is a green stain on the live cells and a red stain on the dead cells. (**B**). Illustrations of the wound area after surgery at various time points. (**C**). The wound size was quantified using a percent-based measurement of wound size compared to the original size. (**D**). Sections of wounds stained with H&E. The red arrows represent the wound length (scale bar = 1000 µm, ∗ *p* < 0.05 comparing duoDERM^®^). Reproduced under the terms of the Creative Commons Attribution 4.0 International (CC BY 4.0) license. [120] Copyright 2021, IOP.

Using peptide-based hydrogels as bioinks, Jang and colleagues published a study in which GelMA hydrogels were combined with VEGF mimicking peptide (Ac-KVKFMDVYQRSYCHP-amide [125]) and then bioprinted [120]. Cell proliferation, viability, and tubular structure formation were stimulated in mouse fibroblasts (NIH 3T3) and HUVECs by VEGF mimicking peptide. Furthermore, this peptide-based hydrogel demonstrated promising wound-healing results in a pig skin wound model (Figure 6). Zhang et al. [121] prepared thermosensitive hydrogels using oyster peptide microspheres as fillers, chitosan as the polymer matrix, and glycerin as the thermal sensitizer. This hydrogel has been demonstrated to accelerate cell migration in L929 cells as a wound-healing hydrogel. As a result of experiments conducted on mouse skin wounds, this hydrogel inhibited the formation of a variety of inflammatory cells, stimulated collagen fiber formation and new blood vessel formation, as well as regulating Ki-67 and VEGF expression in the wound, and enhanced the biosynthesis of total protein in the granulation tissue, leading to rapid wound healing. Furthermore, the hydrogel was non-toxic to L929 cells, making it a promising wound dressing. A thiolated hyaluronic acid-polyethylene diacrylate (tHA-PEGDA) hydrogel containing both immobilized RGD peptides for cell adhesion and anti-VEGF-R2 DNA aptamers for promoting angiogenesis without exogenous growth factors has been developed by Roy and collaborators [122]. Following the reaction of the tHA with the aptamer sequence functionalized with acrylate, the gel was partially crosslinked with PEGDA, and the RGD adhesion peptide was incorporated into it. RGD peptides enhanced cell growth, while the anti-VEGF-R2 DNA aptamer enhanced cell viability as well as angiogenesis by endothelial tube formation, which is helpful in advanced wound healing applications.

## 4. Future Perspective and Challenges

The field of tissue engineering will be further transformed using peptide-based biomaterials since they are engineered to interact more productively with living systems. These materials are inspired by nature as well as a rational design-driven screening and exploration. This emerging technology will be implemented in the clinic more rapidly if these biomaterials are adapted for bioprinting-based biofabrication. Peptide-based hydrogels present challenges and opportunities in tuning their proteolytic stability, specific interactions, and dynamics. For the further development and effectiveness of peptide-based hydrogels, it is crucial to develop methods to increase and improve their stability, as well as increasing the current knowledge about the amino acids present in the peptide structure and interactions. Peptide interactions can be assessed in detail by photo-patterning techniques for enhancement of the spatiotemporal control of biomolecules. The molecular interactions between peptides are physically reversible, making them ideal for injectable biomaterials and bioprinting technologies. Controlling and regulating these interactions can lead to the development of engineered peptide-based bioinks. There is a growing interest in bioprinting and developing peptide-based hydrogels as bioinks. These developments create a promising prospect for the clinical application of these biomaterials. By adjusting the strength of peptide interactions, it is possible to control the dynamics of peptide-based hydrogels. As a result of the dynamicity of the natural ECM, material rearrangements need to be engineered using cell-driven mechanisms on biological timescales. Despite their infancy, stereochemistry-driven interactions between peptides offer a compelling way to tailor peptide assembly stability and thermomechanical properties.

In conclusion, peptide-based hydrogels have been extensively studied for various tissue engineering applications and have recently been developed. Nanofiber peptide-based hydrogels with α-helix and β-sheet content provide the features that scaffolds require from an engineering and biological perspective. Even though this is still a frontier research area, previous publications have provided valuable information. There are, however, three basic steps that should be considered for their development. First, peptide-based hydrogels need to be developed by concentrating on smart hydrogels that are environmentally friendly and can respond to various stimuli. The second step is to study peptide-based multifunctional hydrogels using structural biology and bioinformatics approach. Thirdly, future research and development should focus on the development of bioactive hydrogels based on peptide structures inspired by protein engineering. Increasing and developing theoretical methods have made it easier to study these materials, although further research is required to apply peptide-based hydrogels to clinical trials. By improving their ability to work in the biomedical field, it is hoped that industrial peptide-based hydrogels may prove green and cost-effective in the future.

## Figures and Tables

**Table 1:** A comparison of different types of hydrogels derived from natural resources.

Main Material	Advantages	Disadvantages
Polymers	Biocompatible, Available, Abundance, Easy interaction with cells [63,64]	Need to be manipulated to achieve favorable properties, The synthesis process can affect the final properties, Achieving desired features takes time, Limitations to single-component using [63]
Proteins	Biocompatible, Bioactive [65]- Easy interaction with cells, Similar mechanical, chemical, and structural properties to the ECM, Easy degradation by proteolytic enzymes [66,67]	Proteins must be unfolded to become a hydrogel, Some of physicochemical properties are lost during hydrogelation [56]
Peptides	Biocompatible, Bioactive, The properties of the final hydrogel can be predicted by arranging the appropriate sequence, Easy interaction with cells [68]	Low mechanical properties [69]

**Table 2:** Summary of peptide-based hydrogels for tissue engineering applications.

Target Tissue	Peptides	Other Components	Effects of Peptide	Ref.
Nerve	IKVAV	PNIPAM-b-poly(AC-PEG-COOH) and PNIPAM-b-poly(AC-PEG-IKVAV)	Angiogenic factors expression, Decreasing pro-inflammatory factors, Inhibition of glial scar tissue regrowth	[100]
	RGI and KLT	RADA	Outgrow PC12 cells, Improving the tube-like formation of HUVECs, Facilitation neurovascular crosstalk	[101]
	IKVAV	ssDNA	Cytoskeleton and microtubules differentiation, Longer neurites and modified endocytic mechanisms	[102]
	Ac-RIDARMRADIR-NH2	Glycophorin A (GYPA)	Controlled release of VEGF, Cell transplantation-free regenerative capabilities	[103]
	Fmoc-DIKVAV	-	Promoting cell adhesion and growth, angiogenesis, and neurite outgrowth, Reducing cell death, astrogliosis, Enhancing function in SCI mice.	[104]
	IKVAV-PA and VKIVA-PA	-	Enhancing the function of endogenous or transplanted BMSCs	[105]
Muscle	GGR-KLT	MaHA and Gelatin	Adequate prevention of uncontrolled matrix degradation, Improvements in angiogenesis, Improving myocardial regeneration over 28 days	[106]
	TEMPOL	Polypyrrole	Enhancing cardiomyocytes’ contractile and electrical properties	[107]
	KNRLTIELEVRTC, CIKVAVS, and RKRLQVQLSIRTC	GeG	Differentiation and spreading of C2C12	[108]
	PA	-	Providing myogenic progenitor cells with a suitable biological environment for the survival, maturation, and regeneration of muscle tissue	[109]
Cartilage	RAD-CM	Decellularized Cartilage ECM	Chondrogenic gene expression and ECM deposition	[110]
	NVFFAC	carboxymethyl cellulose dialdehyde and carboxymethyl chitosan	Human chondrocytes were stimulated to undergo in vitro chondrogenesis using hydrogels, and the authors observed enhanced cell growth and cartilage-specific ECM secretion	[111]
	IEIK13	BMP-2, T3	Regeneration of full-thickness cartilage injuries	[112]
	[KLDL]_3_	PDGF-BB, HB-IGF-1	Better integration of newly produced cartilage compared with microfracture alone	[113]
	FKGG-GPQGIWGQ-ERCG	PEG, BMP-2, TGF-3	Formation of osteoblast-like structures and cartilage	[114]
Bone	QK and KP	GelMA, ODex	Considerable increase in the osteogenic differentiation of BMSCs and the angiogenesis capability of HUVECs	[115]
	[COCH3]-RADARADARADARADA-[CONH2]	DEX	Bone tissue regeneration for 21 days with the DEX perfusion rate of 0.1 mL/min	[116]
	RATEA16	rhVEGF165 and BMP-2	Able to deliver growth factors quickly and precisely, Bone regeneration	[117]
	IVFK and IVZK	-	Cell adhesion and spreading of hMSCs	[118]
	FmocFF	HA	Facilitating osteogenic differentiation of MC3T3-E1 preosteoblasts and calcium deposition, Regeneration of ~93% of bone mass	[119]
Skin	Ac-KVKFMDVYQRSYCHP-amide	GelMA, VEGF	Cell proliferation, Viability, Tubular structure formation	[120]
	Oyster	Chitosan, Glycerin	Inhibiting the formation of a variety of inflammatory cells, Stimulating collagen fiber formation and new blood vessel formation, Regulating Ki-67 and VEGF expression in the wound, Enhancing the biosynthesis of total protein in the granulation tissue	[121]
	RGD	tHA-PEGDA, anti-VEGF-R2 DNA	Enhancing cell growth	[122]

## Data Availability

No new data were created or analyzed in this study. Data sharing is not applicable to this article.
